# Restriction of Retroviral Replication by Tetherin/BST-2

**DOI:** 10.1155/2012/424768

**Published:** 2012-07-02

**Authors:** Jason Hammonds, Jaang-Jiun Wang, Paul Spearman

**Affiliations:** Department of Pediatrics, Emory University and Children's Healthcare of Atlanta, 2015 Uppergate Drive, Atlanta, GA 30322, USA

## Abstract

Tetherin/BST-2 is an important host restriction factor that limits the replication of HIV and other enveloped viruses. Tetherin is a type II membrane glycoprotein with a very unusual domain structure that allows it to engage budding virions and retain them on the plasma membrane of infected cells. Following the initial report identifying tetherin as the host cell factor targeted by the HIV-1 Vpu gene, knowledge of the molecular, structural, and cellular biology of tetherin has rapidly advanced. This paper summarizes the discovery and impact of tetherin biology on the HIV field, with a focus on recent advances in understanding its structure and function. The relevance of tetherin to replication and spread of other retroviruses is also reviewed. Tetherin is a unique host restriction factor that is likely to continue to provide new insights into host-virus interactions and illustrates well the varied ways by which host organisms defend against viral pathogens.

## 1. Introduction

 Viruses and their host organisms engage in a series of conflicts in which viruses can be thought of as leading the offense, placing the host on defense. Host defenses against retroviral replication have arisen in a wide variety of forms. Classical cellular and humoral immune responses may limit retroviral replication and may be sufficient to prevent adverse outcomes in some host-virus interactions. However, throughout the evolution of mammals a series of alternative host defense factors have arisen whose apparent primary function is to counteract retroviruses in ways that lie outside of classical innate or adaptive immunity. These intrinsic host defense mechanisms against viruses have come to light largely through comparative studies of inhibition or “restriction” of replication of HIV or SIV in cells from different origins and are collectively referred to as host restriction factors. APOBEC3G, TRIM5alpha, and tetherin are the most prominent of a series of host restriction factors to be identified in recent years that limit HIV replication. This paper focuses on the discovery and subsequent characterization of tetherin, with an emphasis on recent work aimed at elucidating how its structure leads to retention of particles on the plasma membrane and on how Vpu acts to overcome tetherin-mediated restriction.

## 2. Identification of Tetherin as an Antiviral Host Restriction Factor

The discovery of tetherin is intimately linked to studies of the effects of the HIV accessory gene Vpu. Vpu is a small integral membrane protein encoded by HIV-1 and a limited subset of SIV species. Early studies utilizing HIV proviruses deficient for Vpu expression revealed that fewer particles were released from infected cells despite apparently normal production of all other viral proteins [1, 2]. Furthermore, electron microscopic analysis revealed striking accumulations of particles at the cell surface and within intracellular compartments of infected cells, revealing a defect at a late stage of particle release [3]. Subsequent work revealed that one of two important functions of Vpu was the downregulation of CD4 through interactions with cellular proteasomal degradation pathways [4–9]. Vpu was found to bind both CD4 and the human beta transducing-repeat containing protein (*β*-TrCP) [10, 11], connecting CD4 to the ubiquitin-proteasome machinery and inducing its degradation in the endoplasmic reticulum. Casein kinase phosphorylation sites on the Vpu cytoplasmic tail at residues 52 and 56 were found to be critical for *β*-TrCP interactions and for CD4 downregulation [10, 12]. This line of investigation along with other investigations into Vpu function prior to the discovery of tetherin is reviewed in [13]. However, the ability of Vpu to enhance particle release in human cells was not explained by downregulation of CD4 and remained a mystery for many years.

 Experiments leading to the discovery of the function of the HIV Vif protein and its host restriction factor APOBEC3G [14, 15] provided a potential clue to the particle release function of Vpu. Like the infectivity conferred by Vif, the particle release function of Vpu proved to be cell type specific, suggesting that it might be overcoming a cellular factor involved in limiting particle release [16, 17]. A key experiment demonstrated that heterokaryons between restrictive, Vpu-responsive HeLa cells and permissive, Vpu-unresponsive Cos-7 cells were restricted in particle release, suggesting that a negative (restricting) factor was dominant [18]. Vpu was able to enhance particle release in the heterokaryons, demonstrating that the factor from human cells restricting particle release could be overcome by Vpu [18].

 Several cellular factors were described as potential targets of Vpu prior to or concomitant with the identification of tetherin, including TASK-1 [19] and CAML [20]. However, neither of these factors has subsequently proven to be the restriction factor targeted by Vpu. Instead, a series of key findings led by Stuart Neil in the Bieniasz laboratory resulted in the ultimate identification of tetherin as the restriction factor targeted by Vpu. First, these investigators demonstrated clearly that the effect of Vpu was on particle release rather than other steps in virus assembly, while retention of virions and subsequent endocytosis occurred in the absence of Vpu [21]. The specific particle retention activity was found to be prominent in HeLa cells as before, while a subset of human cells such as HOS or 293T cells lacked this activity. The next key observation was that the restricting activity could be induced by type I interferons. Neil and colleagues demonstrated that retention of Vpu-deficient HIV-1 particles at the plasma membrane could be induced in 293T or HOS cells and that treatment with the protease subtilisin released the particles from the cell surface [22]. Furthermore, the restricting activity extended to additional virus genera, as Ebola VP40 release was similarly deficient in an IFN-induced manner and its release could be enhanced by Vpu. These results suggested that an interferon-inducible, proteinaceous tether was responsible for retaining enveloped viruses at the cell surface. In 2008 this factor was identified by the same group as BST-2/CD317 and renamed tetherin because of this prominent biological function [23].

**Figure 1:**
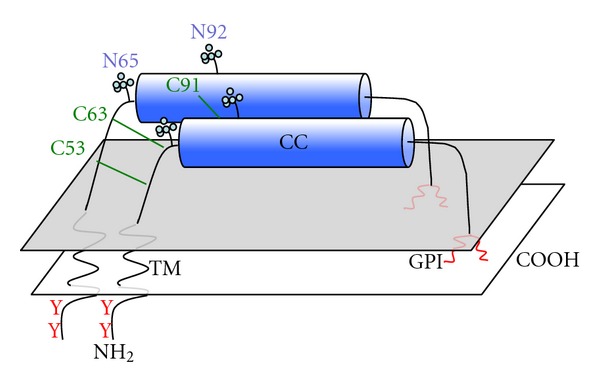
Schematic representation of tetherin domain structure. Tetherin is depicted as a parallel dimer with both transmembrane (TM) and glycophosphatidylinositol (GPI) membrane anchors in the same membrane. Disulfide linkages are depicted in green, and N-linked glycosylation sites pictured. CC: coiled coil; Y: tyrosine residues critical for endocytic motif.

 BST-2 had first been cloned as a membrane antigen present on bone marrow stromal cells and synovial cells that was thought to be involved in pre-B-cell growth [24]. The same protein had been identified as a membrane antigen termed HM1.24, present on terminally differentiated B cells, and was thought to be a potential anticancer target for multiple myeloma [25]. The terminology for the HM1.24 antigen was later changed to CD317 [26]. BST-2 was later shown to be an interferon-inducible antigen and identical to plasmacytoid dendritic cell antigen-1 (PDCA1) in mice [27]. CD317/BST-2 is a highly unusual type II integral membrane protein, with a transmembrane domain near its N-terminus and a C-terminal glycosyl-phosphatidylinositol (GPI) anchor (Figure 1). The protein localizes to lipid rafts on the plasma membrane and to the trans-Golgi network (TGN) and is endocytosed from the plasma membrane through a clathrin-dependent pathway [28]. Remarkably, a membrane proteomic screen examining the effects of the K5 protein of KSHV revealed a marked downregulation of CD317/BST-2 and even showed almost as an afterthought that HIV-1 Vpu downregulated the protein [29]. This published observation led the Guatelli group to examine CD317/BST-2 as a candidate restriction factor targeted by Vpu, and their findings were published soon after the identification of tetherin by the Bieniasz group [30]. For the purpose of this paper, BST-2/CD317/tetherin will be hereafter referred to simply as tetherin.

## 3. Structural Biology of Tetherin and Functional Implications

**Figure 2:**
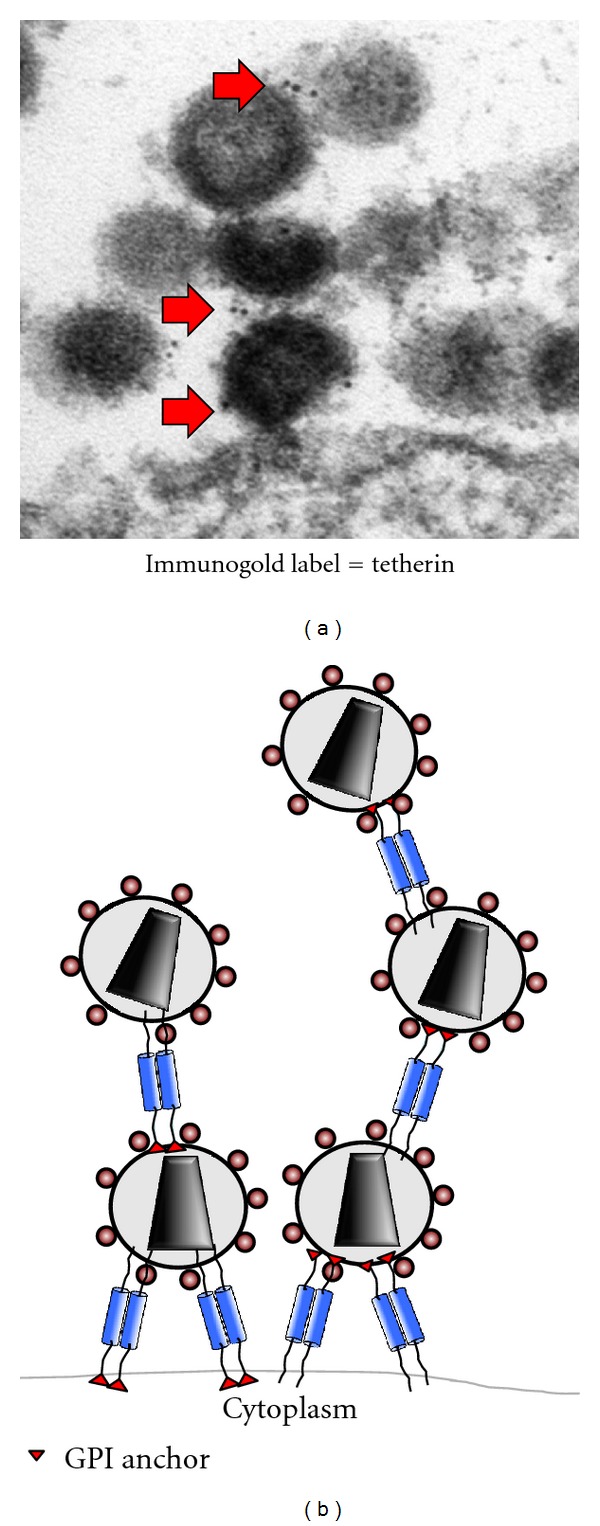
(a) Tetherin on the cell surface of A3.01 T cells infected with NLUdel virus, treated with indinavir to preserve particle morphology for preparation. Arrows indicate immunogold beads; primary antibody was rabbit anti-tetherin polyclonal antisera. (b) Schematic depiction of parallel homodimers of tetherin retaining HIV particles on the plasma membrane; tetherin is not to scale in this diagram.

One of the most fascinating aspects of tetherin biology is how its structure allows for retention of enveloped virions through protein-lipid and protein-protein interactions occurring at the particle budding site. As already mentioned, tetherin's basic domain structure is highly unusual. Tetherin is a type II membrane protein bearing a small N-terminal cytoplasmic domain, a transmembrane region, an ectodomain forming a coiled-coil in tetherin dimers, and a C-terminal GPI anchor (Figure 1) [31]. The double-membrane anchor plays a key role in the ability of tetherin to restrict enveloped virus particle release, presumably because one anchor is present on the plasma membrane of the cell and the second is inserted into the viral membrane [23] (Figure 2). Three cysteines in the N-terminal ectodomain of tetherin (C53, C63, C91) are capable of forming disulfide-linked dimers [32, 33], and mutation of all three abolished dimer formation and greatly reduced the ability of tetherin to restrict Vpu-deficient HIV release [34]. Two N-linked glycosylation sites (N65 and N92) lead to some variability of migration on SDS-PAGE analysis and appear to play a role in correct folding and transport of tetherin to the cell surface in one report [34], while another group found that alteration of N-linked glycosylation sites had no effect on virus restriction or cell surface levels [33].

 Four reports of the tetherin ectodomain structure have been published [35–38]. The ectodomain forms a long extended rod-like conformation in a loose or imperfect coiled-coil parallel dimer [35, 38], suggesting that there is some conformational flexibility in the C-terminal portion of the ectodomain that may be required to accommodate dynamic changes in membrane deformation at the particle budding site. Disulfide bonds stabilize the dimeric N-terminal region, which cannot stably dimerize in their absence [38]. Unexpectedly, tetrameric forms of tetherin were also detected in crystallization studies [36, 38]. The biological function of tetherin tetramers remains uncertain and mutations designed to disrupt the tetramer did not prevent tetherin-mediated particle restriction [36, 38]. The crystal structure of murine BST-2/tetherin ectodomain revealed similar ectodomain architecture, and suggested that tetrameric assemblies may form a curved assembly that functions as a sensor of membrane curvature, analogous to BAR domains [37]. The authors of this paper suggest that tetrameric assemblies may facilitate the clustering of tetherin around the neck of a budding virus as has been seen in immunoelectron microscopic analysis [39, 40]. At the current time, the significance of the tetrameric assemblies remains unclear but quite intriguing.

 While tetherin is thought to be a raft-associated protein through its C-terminal GPI anchor, a recent report questioned this and suggested that instead the C-terminus of tetherin acts as a second transmembrane domain [41]. This unexpected result is intriguing and awaits further verification.

## 4. Tetherin Clustering in Membrane**** Microdomains and Role of the**** Actin Cytoskeleton

 The functional significance of tetherin's unusual structure and topology to its mechanism of restriction of viral budding have not yet been entirely delineated. However, there is significant biochemical and microscopic evidence that tetherin functions as a physical tether connecting virions to the plasma membrane. Immunoelectron microscopic analysis has shown clear evidence of clustering of tetherin on discrete cell surface microdomains and sometimes on filopodia or at the location of coated pits, in the absence of viral infection [39, 40]. In infected cells, immunogold beads are most often observed at the neck of the budding particle and at the site of connections between particle membranes [39, 40] (Figure 2(a)) Tetherin is enriched on the particle membrane itself [39, 40, 42], as well as on filamentous connections that sometimes are present linking particles to one another [40]. Microdomain clustering of tetherin can also be readily observed by superresolution light microscopic techniques [43, 44]. We recently described a tetherin ectodomain mutant with four substitutions in the coiled-coil region (4S) that was expressed well on the cell surface, yet lost the ability to cluster in plasma membrane microdomains and was unable to restrict release of viral particles [43]. The loss of discrete puncta formation of the 4S mutant was associated with an increase in lateral mobility as measured by fluorescence recovery after photobleaching (FRAP), while wild-type, restrictive tetherin was constrained in lateral mobility when compared with classical GPI-anchored proteins [43]. These findings imply that tetherin's restriction of particle release requires localization in discrete microdomains that help to form or are in the immediate vicinity of the developing particle bud. In other words, tetherin's presence on the plasma membrane globally may not be as important as its discrete localization at the site of particle budding. While clustering appears to be associated with restriction, relief of restriction by Vpu is not achieved through removal of tetherin from lipid rafts as measured by partitioning into detergent-resistant membranes [45, 46]. The lack of mobility of tetherin in clustered plasma membrane sites is potentially regulated through interactions not only with lipid microdomains but also with the underlying cytoskeleton.

 The potential for regulation of tetherin clustering through interactions with the underlying actin cytoskeleton is supported by the report from Rollason and colleagues of a direct interaction between tetherin and the RhoGAP protein RICH2 [47]. RICH2 contains both an N-terminal BAR domain and a Rho/Rac/cdc42 GAP domain [48, 49]. The presence of a BAR domain capable of inducing membrane tubulation is curious, given the previously mentioned modeling of tetherin tetramers as a BAR domain [37]. The potential for tetherin to act as a link to the regulation of Rac and Rho through the GAP activity of RICH2 is also intriguing. Perhaps more directly relevant to peripheral clustering of tetherin is the known interaction of RICH2 with EBP50 (ERM-binding phosphoprotein 50) through its C-terminal ESTAL domain [50, 51]. EBP50 acts as a linker between ERM proteins and the cytoplasmic tails of integral membrane proteins, in this case tetherin. This suggests that tetherin is connected indirectly to the underlying cortical actin cytoskeleton through a RICH2-EBP50-ezrin complex. Because RICH2 interacts with the same region of the tetherin cytoplasmic tail that binds *μ*1 and *μ*2 and directs its clathrin-mediated endocytosis [28], the interaction with RICH2 and the actin cytoskeleton might be predicted to stabilize tetherin on the plasma membrane and prevent its endocytosis. Much remains to be learned about the functional role of tetherin's interaction with RICH2 and connection to actin, as well as with the potential modulation of Rho family GTPases. One pressing question that has not yet been addressed is whether this cytoskeletal anchoring plays a role in restriction of particle release and in the punctate clustering of tetherin on the cell surface.

 A counterargument against the role of additional cellular factors in tetherin-mediated restriction may be made in light of evidence from the Bieniasz laboratory demonstrating that an artificial tetherin-like molecule pieced together from domains of three distinct proteins (art-tetherin) can restrict particle release [34]. This strategy employed stitching together the cytoplasmic tail and transmembrane domain of the transferrin receptor, the helical coiled-coil domain of DMPK (dystrophia myotonica protein kinase), and the C-terminus of uPAR that includes a GPI anchor. The investigators in effect recreated the domain architecture of tetherin from sequence-unrelated proteins and quite strikingly were able to inhibit HIV particle release through overexpression of art-tetherin [34]. Despite the ability of this artificial construct to restrict particle release, cellular interactors of wild-type tetherin in relevant human cells clearly play a role in its endocytosis and recycling, and the potential for functional significance of the RICH2-EBP50-ezrin-actin linkage remains.

## 5. Counteraction of Tetherin-Mediated**** Restriction of Particle Release by Vpu

 Following the identification of tetherin as the restriction factor responsible for retention of HIV particles, attention turned to understanding the molecular and cellular mechanisms underlying the relief of tetherin-mediated restriction by Vpu. Comparison of the effects of Vpu on tetherin molecules from nonhuman primates helped to identify critical domains involved in tetherin-Vpu interactions and provided important clues to the evolution of tetherin and of viral countermeasures designed to overcome restriction. Counteraction of tetherin-mediated restriction was mapped to specific interactions between the transmembrane domain of Vpu and the transmembrane domain of tetherin [34, 52–55]. Coimmunoprecipitation studies performed by several groups confirmed a physical interaction between tetherin and Vpu, and the interaction required residues within the TM domains of both Vpu and tetherin as suggested by genetic studies [54, 56–58]. A single-residue alteration in human tetherin to one found in tetherin from the Tantalus monkey (T45I) rendered it Vpu insensitive, yet still able to restrict HIV-1 [55]. Tetherin variants from rhesus macaques and mice were similarly able to restrict HIV-1 release and yet were insensitive to Vpu, and transfer of the corresponding TM region between tetherin molecules from different species conferred sensitivity or resistance [52]. Furthermore, there is strong evidence of positive selection among primate tetherin molecules, and the selected changes were enriched in the N-terminal and TM regions of tetherin, suggesting frequent episodes of evolution under selection pressure to evade viral countermeasures [52, 55]. The discovery that SIV Nef proteins downregulate tetherin from rhesus macaque, sooty mangabey, and African green monkey but are inactive against human tetherin provided evidence that primate lentiviruses have targeted tetherin in different ways over evolutionary history [56, 59]. The Vpu proteins from SIVgsn, SIVmus, and SIVmon are able to downregulate both CD4 and tetherin in cells from their cognate primate species, while Vpu from SIVcpz, the precursor virus of HIV-1, is unable to downregulate chimpanzee tetherin and instead utilizes Nef for this function [60]. The Vpu protein of HIV-1 group M, but not group O or group N, is able to downregulate both tetherin and CD4, and the presence of this fully functional Vpu has been proposed as a reason for the worldwide spread of group M versus the nonpandemic HIV-1 strains [60, 61]. Thus, species-specific differences in tetherin and in lentiviral countermeasures against tetherin have played a major role in cross-species transmission and subsequent spread of lentiviruses and have likely been an important contributor to the current HIV-1 pandemic. While these species-specific differences are the rule, there are exceptions. Shingai and colleagues demonstrated that some HIV-1 Vpu proteins are able to antagonize rhesus tetherin, indicating that some HIV-1 isolates encode a Vpu protein with a broader host range [62].

 Tetherin cell surface levels are downregulated by Vpu, and degradation of tetherin by Vpu has been observed in a wide variety of cell types [30, 54, 63, 64]. The logical hypothesis suggested by this association was that Vpu overcomes restriction by removing tetherin from plasma membrane viral assembly sites and targeting tetherin for degradation, as has been well established for CD4. The downregulation of CD4 by Vpu requires the phosphorylation of serines 52 and 56 on the Vpu cytoplasmic tail, interaction with *β*-TrCP, and degradation of CD4 through the ubiquitin-proteasome pathway [10–12, 65]. The mechanism and importance of downregulation of tetherin by Vpu, however, have not yet been as clearly worked out. Several groups have reported that relief of tetherin-mediated restriction of particle release can occur in the absence of degradation of tetherin [57, 66, 67], indicating that degradation is not the essential step in the action of Vpu that leads to relief of restriction. Goffinet and colleagues generated a series of tetherin cytoplasmic tail mutants including lysine mutants that were not degraded upon expression of Vpu. The mutants remained competent for restriction of particle release, and despite their lack of degradation Vpu potently relieved the restriction to particle release [66]. The involvement of *β*-TrCP in Vpu-mediated targeted degradation of tetherin has been supported by a number of investigators [54, 63, 64, 68], which would seem to suggest that a proteasomal pathway of degradation similar to that involved in the Vpu- *β*-TrCP-CD4 pathway is essential. Proteasomal degradation of tetherin has indeed been supported in some studies [63, 64] but is not universally accepted as the major pathway. Instead, a *β*-TrCP-dependent endolysosomal pathway for tetherin degradation has been reported [54, 58, 68]. According to this model, Vpu still acts as an adaptor molecule linking tetherin to *β*-TrCP, but does not connect tetherin to the ER-associated protein degradation (ERAD) pathway. Instead, interactions in the TGN or early endosome compartments direct tetherin to degradation in lysosomal compartments. There still is work to be done to clarify this pathway and to derive a clearer understanding of the role of *β*-TrCP and of the degradation of tetherin that is initiated or facilitated by Vpu.

 The site of interaction of Vpu with tetherin is not known with certainty. Expression of Vpu alters the intracellular pattern of tetherin, with decreased cell surface of tetherin and prominent colocalization of tetherin and Vpu in the TGN [23, 43, 57, 68]. Mutants of Vpu that are unable to interact with tetherin fail to redistribute tetherin to the TGN, suggesting that tetherin may be retained in the TGN through TM-TM interactions with Vpu [57]. The rate of tetherin endocytosis from the plasma membrane is not significantly altered by Vpu [43, 57, 69]. These data suggest that Vpu may alter delivery of newly synthesized tetherin to the plasma membrane and/or disrupt outward tetherin recycling from the endosomal recycling compartment. Taken together with the data described above regarding endo-lysosomal degradation, a consistent model would posit that Vpu interacts with and traps tetherin in the TGN or other post-ER compartments, thereafter shunting tetherin to degradation in lysosomal compartments and preventing newly synthesized tetherin from trafficking to the plasma membrane. Alternatively, Vpu may disrupt outward trafficking of tetherin to the particle assembly microdomain on the plasma membrane through additional effects on host trafficking factors.

## 6. Counteraction of Tetherin by Other Viruses

 The significance of tetherin as a bona fide host restriction factor is convincingly demonstrated by the fact that diverse families of enveloped viruses have developed distinct mechanisms to overcome its inhibitory effects. One of the earliest factors identified that enhanced the release of *vpu*-deficient HIV-1 and produced efficient release of HIV-2 in restrictive cell types was the envelope glycoprotein of certain strains of HIV-2, in particular ROD10 Env [70–72]. Although the effect of HIV-2 Env on particle release was described well before the identification of tetherin as the target of Vpu, it is now clear that it does so through acting as a tetherin antagonist. HIV-2 Env appears to exclude tetherin from the site of viral budding through direct interaction with tetherin leading to sequestration within the TGN [73]. Determinants of tetherin antagonism by HIV-2 Env include a highly conserved endocytic-sorting motif (GYXX*θ*) in the cytoplasmic tail of gp41 [73, 74]. This sorting motif binds clathrin in an AP-2-dependent manner and is responsible for the redistribution of tetherin from the plasma membrane and concentration within endosomal compartments, in particular the TGN [73, 75, 76]. Interestingly, the gp41 ectodomain of HIV-2 Env has also been implicated in tetherin antagonism [73, 77]. The exact region required for physical tetherin interaction remains unclear due to the inability to differentiate those areas responsible for interaction and those residues involved in maintenance of tertiary Env structure. Additionally, proteolytic Env cleavage into gp120/gp41 subunits is required, as the unprocessed form is incompetent for virion egress and tetherin sequestration [5, 64]. It is interesting to note that, while Vpu expression leads to reduced cellular levels of tetherin, HIV-2 Env reduces cell surface levels but not total cellular levels of tetherin [73]. Finally, the ability of HIV-2 Env to counteract restriction is dependent on conservation of the tetherin ectodomain sequence [78]. Together, these data strongly suggest an interaction between the tetherin and mature HIV-2 Env ectodomains that leads to intracellular trapping of tetherin and abrogates restriction of particle release.

 The K5 protein of KSHV (Human Herpesvirus 8; HHV-8) was the first viral component shown to specifically target tetherin prior to its identification as a viral restriction factor [29]. The K5 protein is a RICH-CH (MARCH) family of cellular transmembrane E3 ubiquitin ligases. This family of proteins facilitates the ubiquitination and subsequent degradation of transmembrane proteins. K5 exhibits potent immunomodulatory function resulting in the degradation of major histocompatibility complex (MHC) proteins (MHC), adhesion molecules, and NK receptor ligands while also promoting the degradation of tetherin through ubiquitination of lysine residues in the tetherin cytoplasmic tail [79, 80]. K5-mediated tetherin degradation is ESCRT-dependent, and ubiquitination of K18 in the CT of tetherin by K5 is critical for the efficient release of KSHV [79, 80]. In the case of K5, it is clear that ubiquitination in a post-ER compartment targets tetherin for degradation via ubiquitin-dependent endolysosomal pathways [80].

 Ebola virus overcomes tetherin-mediated restriction through the activity of its surface glycoprotein (GP) [81]. The Ebola virus GP has a broad species specificity comprising an ability to antagonize both human and murine tetherin. The Ebola GP mechanism of action appears to be novel, as it relieves restriction without reducing tetherin cell surface concentration and can even relieve the restriction conferred by a wholly artificial tetherin molecule [82]. It was recently reported that the GP2 subunit of Ebola interacts with tetherin, and another filovirus GP (Marburg virus GP) was shown to have anti-tetherin activity [83]. The mechanism of action of Ebola GP is perhaps the least clear of the tetherin antagonists that have been described to date.

## 7. *In Vivo* Significance of Tetherin for Viral Spread and Pathogenesis

 The importance of tetherin for restricting viral replication is strongly supported by the multiple mechanisms described above by which viruses can overcome its tethering function and by the evidence of positive selection of tetherin in the primate lineage. The assumption would logically be that tetherin inhibits release of free virus, preventing infection of additional cells and limiting overall replication (and potentially pathogenesis) within an organism. However, whether or not tetherin restricts cell-cell spread remains to be definitively established. Casartelli and coworkers demonstrated that the formation of virologic synapses was not prevented by tetherin, but that tetherin did limit cell-cell transmission of virus [84]. Another group found similarly that cell-cell transmission was inhibited by tetherin in a flow-cytometry-based assay [85]. In contrast, Jolly and colleagues demonstrated that depletion of tetherin diminished virologic synapse formation and cell-cell spread and suggested that under some circumstances tetherin may actually enhance cell-cell transmission [86]. Depletion of tetherin in mature dendritic cells was not associated with a significant enhancement of transmission to CD4+ T cells in another report, although modest enhancement or inhibition of cell-cell transmission was seen that differed with the stimulus utilized for maturation of dendritic cells [87]. Currently there is a need for further investigation into this question, as there is not a clear consensus in the field.

 Tetherin knockout mice have provided additional weight to the argument that this protein has evolved as an interferon-induced host defense mechanism to limit viral replication *in vivo*. Liberatore and Bieniasz used poly(I : C) to enhance tetherin expression in wild-type mice and found that replication of Moloney murine leukemia virus (Mo-MLV) in these mice was significantly attenuated as compared with tetherin-deficient mice [88]. Using a murine leukemia virus strain that induces a strong interferon response, they then demonstrated that tetherin-deficient mice developed both higher levels of MLV viremia and enhanced pathology [88]. A different strategy utilizing a naturally occurring polymorphism in tetherin in NZW mice allowed Barrett and colleagues to study Friend virus replication in mice homozygous for enhanced versus normal tetherin cell surface expression. These investigators demonstrated that enhanced cell surface tetherin *in vivo* correlated with diminished replication of Friend virus and improved outcomes [89]. Together these reports provide solid evidence that tetherin acts as an antiretroviral host restriction factor *in vivo*. A modest inhibitory effect of tetherin on Mo-MLV replication was also reported by Swiecki and colleagues, consistent with the effects seen by Liberatore and Bieniasz in the absence of IFN induction [90]. Surprisingly, however, these authors observed lower viral titers and enhanced virus-specific CD8+ T-cell responses in tetherin-deficient mice infected with vesicular stomatitis virus or influenza virus. Thus, while tetherin's antiretroviral effects are clear, there may be more complexity in how tetherin alters antigen processing and affects the replication of other enveloped viruses *in vivo*.

## 8. Summary

 Tetherin is an unusual host protein that restricts enveloped particle release at the very latest stage of the viral lifecycle through physically tethering virions to the plasma membrane. A number of unrelated viruses have developed the means to overcome restriction by tetherin and have done so through different mechanisms. The acquisition of Vpu by primate lentiviruses and its ability to counteract restriction by human tetherin is thought to be an important factor in cross-species transmission and potentially in the magnitude of the HIV-1 pandemic itself. The flurry of recent studies examining tetherin and its antagonists emphasizes the significance of this potent antiviral host restriction factor. Future studies should shed light not only on the mechanism of action of Vpu, but will likely identify additional enveloped viruses that have developed the means to antagonize tetherin. Studies examining the cellular interactions of tetherin are also poised to provide new insights into the nature of the particle assembly site, trafficking of membrane glycoproteins to the particle assembly site, and the role of the cortical actin cytoskeleton in particle release.

## References

[1] Strebel K, Klimkait T, Martin MA (1988). A novel gene of HIV-1, vpu, and its 16-kilodalton product. *Science*.

[2] Terwilliger EF, Godin B, Sodroski JG, Haseltine WA (1989). Construction and use of a replication-competent human immunodeficiency virus (HIV-1) that expresses the chloramphenicol acetyltransferase enzyme. *Proceedings of the National Academy of Sciences of the United States of America*.

[3] Klimkait T, Strebel K, Hoggan MD, Martin MA, Orenstein JM (1990). The human immunodeficiency virus type 1-specific protein vpu is required for efficient virus maturation and release. *Journal of Virology*.

[4] Willey RL, Buckler-White A, Strebel K (1994). Sequences present in the cytoplasmic domain of CD4 are necessary and sufficient to confer sensitivity to the human immunodeficiency virus type 1 Vpu protein. *Journal of Virology*.

[5] Lenburg ME, Landau NR (1993). Vpu-induced degradation of CD4: requirement for specific amino acid residues in the cytoplasmic domain of CD4. *Journal of Virology*.

[6] Vincent MJ, Raja NU, Jabbar MA (1993). Human immunodeficiency virus type 1 Vpu protein induces degradation of chimeric envelope glycoproteins bearing the cytoplasmic and anchor domains of CD4: role of the cytoplasmic domain in Vpu-induced degradation in the endoplasmic reticulum. *Journal of Virology*.

[7] Geraghty RJ, Panganiban AT (1993). Human immunodeficiency virus type 1 Vpu has a CD4- and an envelope glycoprotein-independent function. *Journal of Virology*.

[8] Chen MY, Maldarelli F, Karczewski MK, Willey RL, Strebel K (1993). Human immunodeficiency virus type 1 Vpu protein induces degradation of CD4 in vitro: the cytoplasmic domain of CD4 contributes to Vpu sensitivity. *Journal of Virology*.

[9] Willey RL, Maldarelli F, Martin MA, Strebel K (1992). Human immunodeficiency virus type 1 Vpu protein induces rapid degradation of CD4. *Journal of Virology*.

[10] Margottin F, Bour SP, Durand H (1998). A novel human WD protein, h-*β*TrCP, that interacts with HIV-1 Vpu connects CD4 to the ER degradation pathway through an F-box motif. *Molecular Cell*.

[11] Schubert U, Antón LC, Bačík I (1998). CD4 glycoprotein degradation induced by human immunodeficiency virus type 1 Vpu protein requires the function of proteasomes and the ubiquitin- conjugating pathway. *Journal of Virology*.

[12] Paul M, Jabbar MA (1997). Phosphorylation of both phosphoacceptor sites in the HIV-1 Vpu cytoplasmic domain is essential for Vpu-mediated ER degradation of CD4. *Virology*.

[13] Bour S, Strebel K (2003). The HIV-1 Vpu protein: a multifunctional enhancer of viral particle release. *Microbes and Infection*.

[14] Sheehy AM, Gaddis NC, Choi JD, Malim MH (2002). Isolation of a human gene that inhibits HIV-1 infection and is suppressed by the viral Vif protein. *Nature*.

[15] Simon JHM, Miller DL, Fouchier RAM, Soares MA, Peden KWC, Malim MH (1998). The regulation of primate immunodeficiency virus infectivity by Vif is cell species restricted: a role for Vif in determining virus host range and cross-species transmission. *The EMBO Journal*.

[16] Geraghty RJ, Talbot KJ, Callahan M, Harper W, Panganiban AT (1994). Cell type-dependence for Vpu function. *Journal of Medical Primatology*.

[17] Sakai H, Tokunaga K, Kawamura M, Adachi A (1995). Function of human immunodeficiency virus type 1 Vpu protein in various cell types. *Journal of General Virology*.

[18] Varthakavi V, Smith RM, Bour SP, Strebel K, Spearman P (2003). Viral protein U counteracts a human host cell restriction that inhibits HIV-1 particle production. *Proceedings of the National Academy of Sciences of the United States of America*.

[19] Hsu K, Seharaseyon J, Dong P, Bour S, Marbán E (2004). Mutual functional destruction of HIV-1 Vpu and host TASK-1 channel. *Molecular Cell*.

[20] Varthakavi V, Heimann-Nichols E, Smith RM (2008). Identification of calcium-modulating cyclophilin ligand as a human host restriction to HIV-1 release overcome by Vpu. *Nature Medicine*.

[21] Neil SJ, Eastman SW, Jouvenet N, Bieniasz PD (2006). HIV-1 Vpu promotes release and prevents endocytosis of nascent retrovirus particles from the plasma membrane. *PLoS Pathogens*.

[22] Neil SJD, Sandrin V, Sundquist WI, Bieniasz PD (2007). An interferon-alpha-induced tethering mechanism inhibits HIV-1 and Ebola virus particle release but is counteracted by the HIV-1 Vpu protein. *Cell Host and Microbe*.

[23] Neil SJD, Zang T, Bieniasz PD (2008). Tetherin inhibits retrovirus release and is antagonized by HIV-1 Vpu. *Nature*.

[24] Ishikawa J, Kaisho T, Tomizawa H (1995). Molecular cloning and chromosomal mapping of a bone marrow stromal cell surface gene, BST2, that may be involved in pre-B-cell growth. *Genomics*.

[25] Goto T, Kennel SJ, Abe M (1994). A novel membrane antigen selectively expressed on terminally differentiated human B cells. *Blood*.

[26] Vidal-Laliena M, Romero X, March S, Requena V, Petriz J, Engel P (2005). Characterization of antibodies submitted to the B cell section of the 8th Human Leukocyte Differentiation Antigens Workshop by flow cytometry and immunohistochemistry. *Cellular Immunology*.

[27] Blasius AL, Giurisato E, Cella M, Schreiber RD, Shaw AS, Colonna M (2006). Bone marrow stromal cell antigen 2 is a specific marker of type I IFN-producing cells in the naive mouse, but a promiscuous cell surface antigen following IFN stimulation. *Journal of Immunology*.

[28] Rollason R, Korolchuk V, Hamilton C, Schu P, Banting G (2007). Clathrin-mediated endocytosis of a lipid-raft-associated protein is mediated through a dual tyrosine motif. *Journal of Cell Science*.

[29] Bartee E, McCormack A, Früh K (2006). Quantitative membrane proteomics reveals new cellular targets of viral immune modulators. *PLoS Pathogens*.

[30] Van Damme N, Goff D, Katsura C (2008). The interferon-induced protein BST-2 restricts HIV-1 release and is downregulated from the cell surface by the viral Vpu protein. *Cell Host and Microbe*.

[31] Kupzig S, Korolchuk V, Rollason R, Sugden A, Wilde A, Banting G (2003). Bst-2/HM1.24 is a raft-associated apical membrane protein with an unusual topology. *Traffic*.

[32] Ohtomo T, Sugamata Y, Ozaki Y (1999). Molecular cloning and characterization of a surface antigen preferentially overexpressed on multiple myeloma cells. *Biochemical and Biophysical Research Communications*.

[33] Andrew AJ, Miyagi E, Kao S, Strebel K (2009). The formation of cysteine-linked dimers of BST-2/tetherin is important for inhibition of HIV-1 virus release but not for sensitivity to Vpu. *Retrovirology*.

[34] Perez-Caballero D, Zang T, Ebrahimi A (2009). Tetherin inhibits HIV-1 release by directly tethering virions to cells. *Cell*.

[35] Hinz A, Miguet N, Natrajan G (2010). Structural basis of HIV-1 tethering to membranes by the BST-2/tetherin ectodomain. *Cell Host and Microbe*.

[36] Schubert HL, Zhai Q, Sandrin V (2010). Structural and functional studies on the extracellular domain of BST2/tetherin in reduced and oxidized conformations. *Proceedings of the National Academy of Sciences of the United States of America*.

[37] Swiecki M, Scheaffer SM, Allaire M, Fremont DH, Colonna M, Brett TJ (2011). Structural and biophysical analysis of BST-2/tetherin ectodomains reveals an evolutionary conserved design to inhibit virus release. *The Journal of Biological Chemistry*.

[38] Yang H, Wang J, Jia X (2010). Structural insight into the mechanisms of enveloped virus tethering by tetherin. *Proceedings of the National Academy of Sciences of the United States of America*.

[39] Fitzpatrick K, Skasko M, Deerinck TJ, Crum J, Ellisman MH, Guatelli J (2010). Direct restriction of virus release and incorporation of the interferon-induced protein BST-2 into HIV-1 particles. *PLoS Pathogens*.

[40] Hammonds J, Wang JJ, Yi H, Spearman P (2010). Immunoelectron microscopic evidence for tetherin/BST2 as the physical bridge between HIV-1 virions and the plasma membrane. *PLoS Pathogens*.

[41] Andrew AJ, Kao S, Strebel K (2011). C-terminal hydrophobic region in human bone marrow stromal cell antigen 2 (BST-2)/tetherin protein functions as second transmembrane motif. *The Journal of Biological Chemistry*.

[42] Habermann A, Krijnse-Locker J, Oberwinkler H (2010). CD317/tetherin is enriched in the HIV-1 envelope and downregulated from the plasma membrane upon virus infection. *Journal of Virology*.

[43] Hammonds J, Ding L, Chu H (2012). The tetherin/BST-2 coiled-coil ectodomain mediates plasma membrane microdomain localization and restriction of particle release. *Journal of Virology*.

[44] Lehmann M, Rocha S, Mangeat B (2011). Quantitative multicolor super-resolution microscopy reveals tetherin HIV-1 interaction. *PLoS Pathogens*.

[45] Lopez LA, Yang SJ, Exline CM, Rengarajan S, Haworth KG, Cannon PM (2012). Anti-tetherin activities of HIV-1 Vpu and ebola virus glycoprotein do not involve removal of tetherin from lipid rafts. *Journal of Virology*.

[46] Fritz JV, Tibroni N, Keppler OT, Fackler OT (2012). HIV-1 Vpu's lipid raft association is dispensable for counteraction of the particle release restriction imposed by CD317/Tetherin. *Virology*.

[47] Rollason R, Korolchuk V, Hamilton C, Jepson M, Banting G (2009). A CD317/tetherin-RICH2 complex plays a critical role in the organization of the subapical actin cytoskeleton in polarized epithelial cells. *The Journal of Cell Biology*.

[48] Katoh Y, Katoh M (2004). Identification and characterization of ARHGAP27 gene in silico. *International Journal of Molecular Medicine*.

[49] Richnau N, Aspenström P (2001). Rich, a rho GTPase-activating protein domain-containing protein involved in signaling by Cdc42 and Rac1. *The Journal of Biological Chemistry*.

[50] Reczek D, Bretscher A (2001). Identification of EPI64, a TBC/rabGAP domain-containing microvillar protein that binds to the first PDZ domain of EBP50 and E3KARP. *Journal of Cell Biology*.

[51] Songyang Z, Shoelson SE, Chaudhuri M (1993). SH2 domains recognize specific phosphopeptide sequences. *Cell*.

[52] McNatt MW, Zang T, Hatziioannou T (2009). Species-specific activity of HIV-1 Vpu and positive selection of tetherin transmembrane domain variants. *PLoS Pathogens*.

[53] Rong L, Zhang J, Lu J (2009). The transmembrane domain of BST-2 determines its sensitivity to down-modulation by human immunodeficiency virus type 1 Vpu. *Journal of Virology*.

[54] Douglas JL, Viswanathan K, McCarroll MN, Gustin JK, Früh K, Moses AV (2009). Vpu directs the degradation of the human immunodeficiency virus restriction factor BST-2/tetherin via a *β*TrCP-dependent mechanism. *Journal of Virology*.

[55] Gupta RK, Hué S, Schaller T, Verschoor E, Pillay D, Towers GJ (2009). Mutation of a single residue renders human tetherin resistant to HIV-1 Vpu-mediated depletion. *PLoS Pathogens*.

[56] Jia B, Serra-Moreno R, Neidermyer W (2009). Species-specific activity of SIV Nef and HIV-1 Vpu in overcoming restriction by tetherin/BST2. *PLoS Pathogens*.

[57] Dubé M, Roy BB, Guiot-Guillain P (2010). Antagonism of tetherin restriction of HIV-1 release by Vpu involves binding and sequestration of the restriction factor in a perinuclear compartment. *PLoS Pathogens*.

[58] Iwabu Y, Fujita H, Kinomoto M (2009). HIV-1 accessory protein Vpu internalizes cell-surface BST-2/tetherin through transmembrane interactions leading to lysosomes. *The Journal of Biological Chemistry*.

[59] Zhang F, Wilson SJ, Landford WC (2009). Nef proteins from simian immunodeficiency viruses are tetherin antagonists. *Cell Host and Microbe*.

[60] Sauter D, Schindler M, Specht A (2009). Tetherin-driven adaptation of Vpu and Nef function and the evolution of pandemic and nonpandemic HIV-1 strains. *Cell Host and Microbe*.

[61] Gupta RK, Towers GJ (2009). A tail of Tetherin: how pandemic HIV-1 conquered the world. *Cell Host and Microbe*.

[62] Shingai M, Yoshida T, Martin MA, Strebel K (2011). Some human immunodeficiency virus type 1 Vpu proteins are able to antagonize macaque BST-2 In Vitro and In vivo: Vpu-Negative simian-human immunodeficiency viruses are attenuated In vivo. *Journal of Virology*.

[63] Goffinet C, Allespach I, Homann S (2009). HIV-1 antagonism of CD317 is species specific and involves Vpu-mediated proteasomal degradation of the restriction factor. *Cell Host and Microbe*.

[64] Mangeat B, Gers-Huber G, Lehmann M, Zufferey M, Luban J, Piguet V (2009). HIV-1 Vpu neutralizes the antiviral factor tetherin/BST-2 by binding it and directing its beta-TrCP2-dependent degradation. *PLoS Pathogens*.

[65] Margottin F, Benichou S, Durand H (1996). Interaction between the cytoplasmic domains of HIV-1 Vpu and CD4: role of Vpu residues involved in CD4 interaction and in vitro CD4 degradation. *Virology*.

[66] Goffinet C, Homann S, Ambiel I (2010). Antagonism of CD317 restriction of human immunodeficiency virus type 1 (HIV-1) particle release and depletion of CD317 are separable activities of HIV-1 Vpu. *Journal of Virology*.

[67] Miyagi E, Andrew AJ, Kao S, Strebe K (2009). Vpu enhances HIV-1 virus release in the absence of Bst-2 cell surface down-modulation and intracellular depletion. *Proceedings of the National Academy of Sciences of the United States of America*.

[68] Mitchell RS, Katsura C, Skasko MA (2009). Vpu antagonizes BST-2-mediated restriction of HIV-1 release via *β*-TrCP and endo-lysosomal trafficking. *PLoS Pathogens*.

[69] Andrew AJ, Miyagi E, Strebel K (2011). Differential effects of human immunodeficiency virus type 1 Vpu on the stability of BST-2/tetherin. *Journal of Virology*.

[70] Bour S, Schubert U, Peden K, Strebel K (1996). The envelope glycoprotein of human immunodeficiency virus type 2 enhances viral particle release: a Vpu-like factor?. *Journal of Virology*.

[71] Bour S, Strebel K (1996). The human immunodeficiency virus (HIV) type 2 envelope protein is a functional complement to HIV type 1 Vpu that enhances particle release of heterologous retroviruses. *Journal of Virology*.

[72] Ritter GD, Yamshchikov G, Cohen SJ, Mulligan MJ (1996). Human immunodeficiency virus type 2 glycoprotein enhancement of particle budding: role of the cytoplasmic domain. *Journal of Virology*.

[73] Le Tortorec A, Neil SJD (2009). Antagonism to and intracellular sequestration of human tetherin by the human immunodeficiency virus type 2 envelope glycoprotein. *Journal of Virology*.

[74] Abada P, Noble B, Cannon PM (2005). Functional domains within the human immunodeficiency virus type 2 envelope protein required to enhance virus production. *Journal of Virology*.

[75] Hauser H, Lopez LA, Yang SJ (2010). HIV-1 Vpu and HIV-2 Env counteract BST-2/tetherin by sequestration in a perinuclear compartment. *Retrovirology*.

[76] Noble B, Abada P, Nunez-Iglesias J, Cannon PM (2006). Recruitment of the adaptor protein 2 complex by the human immunodeficiency virus type 2 envelope protein is necessary for high levels of virus release. *Journal of Virology*.

[77] Bour S, Akari H, Miyagi E, Strebel K (2003). Naturally occurring amino acid substitutions in the HIV-2 ROD envelope glycoprotein regulate its ability to augment viral particle release. *Virology*.

[78] Gupta RK, Mlcochova P, Pelchen-Matthews A (2009). Simian immunodeficiency virus envelope glycoprotein counteracts tetherin/BST-2/CD317 by intracellular sequestration. *Proceedings of the National Academy of Sciences of the United States of America*.

[79] Mansouri M, Viswanathan K, Douglas JL (2009). Molecular mechanism of BST2/tetherin downregulation by K5/MIR2 of Kaposi’s sarcoma-associated herpesvirus. *Journal of Virology*.

[80] Pardieu C, Vigan R, Wilson SJ (2010). The RING-CH ligase K5 antagonizes restriction of KSHV and HIV-1 particle release by mediating ubiquitin-dependent endosomal degradation of tetherin. *PLoS Pathogens*.

[81] Bates P, Kaletsky RL, Francica JR, Agrawal-Gamse C (2009). Tetherin-mediated restriction of filovirus budding is antagonized by the Ebola glycoprotein. *Proceedings of the National Academy of Sciences of the United States of America*.

[82] Lopez LA, Yang SJ, Hauser H (2010). Ebola virus glycoprotein counteracts BST-2/tetherin restriction in a sequence-independent manner that does not require tetherin surface removal. *Journal of Virology*.

[83] Kühl A, Banning C, Marzi A (2011). The Ebola virus glycoprotein and HIV-1 VPU employ different strategies to counteract the antiviral factor tetherin. *Journal of Infectious Diseases*.

[84] Casartelli N, Sourisseau M, Feldmann J (2010). Tetherin restricts productive HIV-1 cell-to-cell transmission. *PLoS Pathogens*.

[85] Kuhl BD, Sloan RD, Donahue DA, Bar-Magen T, Liang C, Wainberg MA (2010). Tetherin restricts direct cell-to-cell infection of HIV-1. *Retrovirology*.

[86] Jolly C, Booth NJ, Neil SJD (2010). Cell-cell spread of human immunodeficiency virus type 1 overcomes tetherin/BST-2-mediated restriction in T cells. *Journal of Virology*.

[87] Coleman CM, Spearman P, Wu L (2011). Tetherin does not significantly restrict dendritic cell-mediated HIV-1 transmission and its expression is upregulated by newly synthesized HIV-1 Nef. *Retrovirology*.

[88] Liberatore RA, Bieniasz PD (2011). Tetherin is a key effector of the antiretroviral activity of type I interferon in vitro and in vivo. *Proceedings of the National Academy of Sciences of the United States of America*.

[89] Barrett BS, Smith DS, Li SX, Guo K, Hasenkrug KJ, Santiago ML (2012). A single nucleotide polymorphism in tetherin promotes retrovirus restriction in vivo. *PLoS Pathogens*.

[90] Swiecki M, Wang Y, Gilfillan S, Lenschow DJ, Colonna M (2012). Cutting edge: paradoxical roles of BST2/tetherin in promoting type I IFN response and viral infection. *Journal of Immunology*.

